# A Statistical Evolution Model of Concrete Damage Induced by Seawater Corrosion

**DOI:** 10.3390/ma14041007

**Published:** 2021-02-20

**Authors:** Hangjie Lv, Jiankang Chen, Chunsheng Lu

**Affiliations:** 1 Key Laboratory of Impact and Safety Engineering, School of Mechanical Engineering and Mechanics, Ningbo University, Ningbo 315211, China; 1811081010@nbu.edu.cn; 2 School of Civil and Mechanical Engineering, Curtin University, Perth, WA 6845, Australia; C.Lu@curtin.edu.au

**Keywords:** chemical corrosion, corrosion damage, concrete, statistical evolution, micro-cracks

## Abstract

The transmission of sulfate ions in concrete results in formation of calcium sulfoaluminate crystals due to chemical reactions. The expansion of calcium sulfoaluminate crystals is the main cause of concrete corrosion damage. In this study, ultrasonic analysis was used to detect the modulus change of concrete due to sulfate corrosion to obtain the basic law of corrosion damage evolution. An exponential growth model was developed for the internal expansion force based on the chemical reaction rate of calcium sulfoaluminate crystallization. Then, the evolution equation of the number density of microcracks was derived based on their initiation and balance conditions. Finally, a statistical model was developed for the concrete damage evolution by integrating the volume of microcracks. It is shown that the statistical evolution model can well characterize the evolution of concrete corrosion damage.

## 1. Introduction

It is well-known that seawater contains a large number of ions that are harmful to concrete, among which sulfate corrosion is one of the most important mechanisms for degradation of concrete’s durability in marine environments [1,2,3]. Expansive stress could be generated due to the chemical reactions between diffused sulfate ions and the pore solution in concrete structures. The stress significantly affects the mechanical properties and durability of cement-based materials [4,5]. The nucleation and growth of microcracks might occur under the action of an internal expansive stress and an external load, which ultimately decreases the service life of concrete structures.

The majority of the previous research on the durability of concrete structures in marine environments has focused on corrosion due to chloride ions [6,7,8]. Sulfate attack not only induces damage in concrete in marine environments and diminishes its mechanical properties [9], but also supplies more paths for the diffusion of chloride ions. Therefore, sulfate attack on concrete has been one of the most important research subjects in recent decades. Although the harmful effects of sulfate corrosion on concrete structures have been realized for some time, due to the complexity of the problem, relevant research on the corrosion mechanism and damage evolution has been conducted only during the past 20 years; for instance, Adam [10] suggested that sulfate attack on concrete was a “confused word”.

Ettringite and gypsum are the major products of sulfate attack that cause expansion of concrete, although the exact mechanism is not fully understood [11,12,13]. Currently, the most commonly used theories include swelling [14], topological chemical reaction [15], and crystal growth pressure [16,17]. When sulfate ions are diffused in concrete pores, a chemical equilibrium is disturbed between the solid phase and interstitial solution in the cemented matrix [18,19,20]. Chemical reactions between ions and pore solution produce gypsum, thaumasite, and delayed ettringite [21,22,23]. As the corrosion age increases, reaction products gradually accumulate in the pores or interfacial transition zones of concrete, resulting in generation of expansion pressure. In turn, this expansion pressure generates tensile stress. If the stress exceeds the tensile strength of the concrete structure, microcracks nucleate. The evolution of corrosion cracks diminishes the concrete’s density and porosity, and this leads to deterioration of the concrete [24]. Because corrosion damage can decrease concrete durability, there have been many studies on this topic. For example, Monteiro and Kurtis [25] studied the time of expansion failure of concrete specimens immersed in sulfate solution. Yang et al. [26] investigated the influence of sulfate attack on variations of the concrete modulus. Wardeh and Toutanji [27] combined elastic and continuum damage mechanics to develop an anisotropic damage model for concrete.

To accurately evaluate the service life of concrete structures under sulfate attack, researchers have proposed several damage models based on variation of the macroscopic mechanical properties of concrete structures. To develop the STADIUM (stands for Software for Transport and Degradation In (Un)saturated Materials) model for sulfate corrosion, Marchand et al. [28] evaluated the effects of various cement types, corrosion solution concentrations, and water–cement ratios on corrosion damage. Sarkar et al. [29] established a mechanical damage model based on the STADIUM model. In this modified model, the process of concrete failure was described in detail and the stress–strain curve of concrete specimens was obtained under sulfate attack. These damage models based on the macroscopic mechanical properties of concrete are valid for the evaluation of concrete damage. Regarding the mesoscopic structure of concrete, however, meso-mechanical models must be based on the meso-physical mechanism of material damage. Thus, the damage effect on the macro properties of materials should be taken into account. The relationship between the macroscopic mechanical behaviors of materials and damage parameters under sulfate attack is of significant importance. Although a macroscopic mechanical property-based concrete damage model is important in damage evaluation, establishing a meso-mechanics model based on the micro-structure of concrete is more reasonable. Researchers have developed several meso-mechanics [30], grid [31], and random aggregate models [32] for evaluation of damage and fracture processes of concrete. As an example, Basista and Weglewski [33] proposed a micromechanical model for deformation simulation of cementitious composites due to external sulfate attack.

In contrast, few reports have been published on the damage evolution of microcracks due to sulfate attack. Microcracks generated by corrosion can be expanded and converged until concrete failure. In addition, macro-damage in materials generated by accumulation of micro-damages may have a catastrophic consequence. In this study, the evolution of corrosion damage of concrete due to sulfate attack was investigated by statistical method, in which the effect of chemical reaction rate was taken into account. Then, a statistical evolution model of corrosion damage of concrete was developed. Results showed the proposed model can well describe the characteristics of corrosion damage evolution.

## 2. Mathematical Characterization of Corrosion Damage Evolution

The chemical reaction takes place during diffusion of sulfate ions in concrete. The main chemical process includes [34]:

(1) $$CH+SO_{4}^{2-}\to C\bar{S}H_{2}+2OH^{-}$$

and:

(2) $$\begin{array}{l} CA+qCSH_{2}\to C_{6}A{\bar{S}}_{3}H_{32}\\ CA:=\gamma_{1}C_{3}A+\gamma_{2}C_{4}AH_{13}+\gamma_{3}C_{4}A\bar{S}H_{12}\\ q=3\gamma_{1}+3\gamma_{2}+2\gamma_{3} \end{array}$$

where CH and $C\bar{S}H_{2}$, respectively, denote calcium hydroxide and gypsum; symbols C_4_AH_13_ and $C_{4}A\bar{S}H_{12}$, respectively, represent tetracalcium aluminate and monosulfate; *q* is the stoichiometric weighted coefficient of the sulfate phase; *γ*_i_ (i = 1–3) denotes the proportion of each aluminate phase to the total aluminate content.

The chemical reaction rate equation can be analytically obtained from Equations (1) and (2), that is:

(3) $$\frac{dC_{CA}}{dt}=-\frac{kC_{CA}C_{SO}}{q}\;\frac{dC_{SO}}{dt}=-kC_{CA}C_{SO}$$

where $C_{CA}$ and $C_{SO}$ are the concentrations of CA and sulfate ions, and k is the coefficient of reaction. Then, we have:

(4) $$\frac{dC_{SO}}{dt}=q\frac{dC_{CA}}{dt}$$

From Equations (3) and (4) we can obtain:

(5) $$\frac{dC_{CA}}{dt}=-kC_{CA}(C_{CA}+\beta_{p})$$

Usually, $C_{CA}$ is much less than 1, thus the second order small quantity on the right-hand side of Equation (5) can be neglected; we obtained:

(6) $$\frac{dC_{CA}}{dt}=-k\beta_{p}C_{CA}$$

As shown in the first formula of Equation (2), the increase in ettringite concentration, $dC_{Ettringite}$, is proportional to the decrease in concentration, $dC_{CA}$, namely:

(7) $$dC_{Ettringite}\propto -dC_{CA}$$

**Figure 1:**
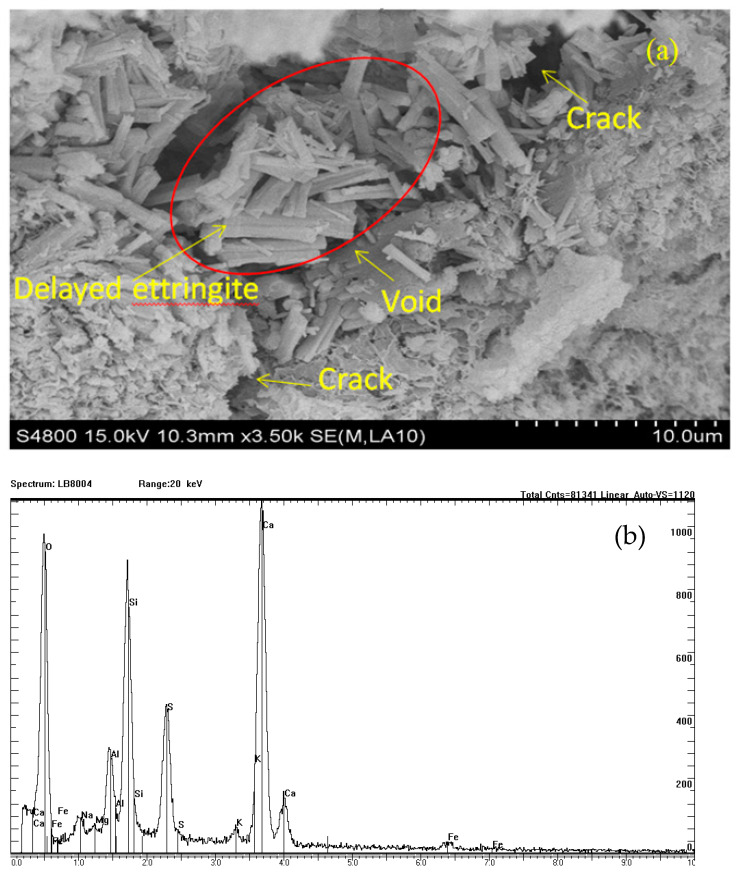
(**a**) SEM image of damage in a concrete sample immersed in sodium sulfate solution with 8% concentration for 250 days, and (**b**) the energy spectrum of ettingite.

Generally speaking, the expansive stress appears due to growth of ettringite in pores of concrete. This stress leads to nucleation and growth of microcracks in concrete materials, as observed in Figure 1, where the main crack area is indicated by arrows in the lower-left and upper-right corners. Due to the corrosion effect of sodium sulfate solution, ettringite grew in the crack, with the columnar crystal representing delayed ettringite formation. Due to the expansion effect of ettringite, the main crack area continuously expanded and another crack was generated along the internal expansion direction. This suggests that concrete damage may be caused by expansion stress produced by delayed ettringite.

Here let us assume that the number density of microcracks in concrete at time *t* of service is [35,36]:

(8) n=n(t, a)

The definition given by Equation (1) means the number of cracks with length [*a*, *a* + d*a*] perunit volume is *n*(*t*, *a*)d*a*.

Chen et al. [37] proposed an empirical formula to describe the corrosion damage evolution. The damage degree *D* is defined as the volume occupied by microcracks per unit volume. Therefore, *D* can be considered as the sum of all crack volumes in a unit volume of concrete, that is:

(9) $$D=\int_{0}^{\infty }n\left(t,\;a\right)\beta_{a}a^{3}da$$

where *β_a_* is a dimensionless parameter.

**Figure 2:**
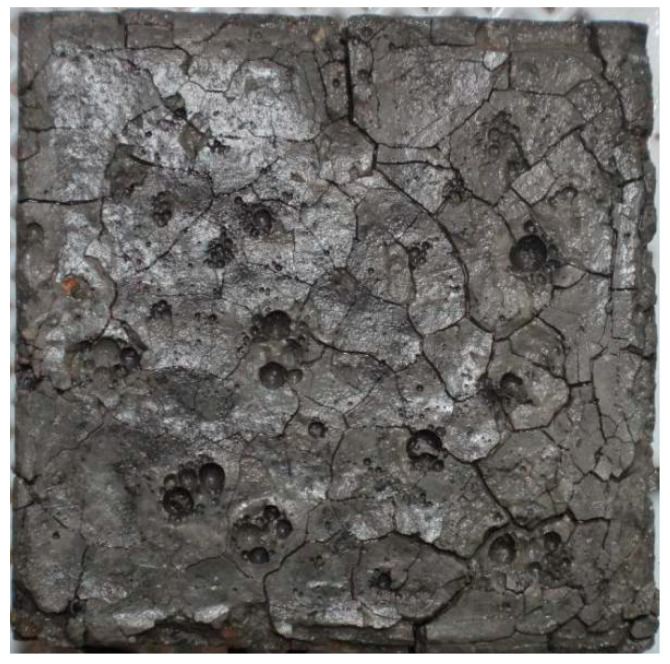
Corrosion cracks on surface of a concrete sample immersed in sodium sulfate solution of 8% concentration for 250 days.

Concrete degradation due to corrosion mainly includes the nucleation, growth, and convergence of microcracks (see Figure 2). However, macrocracks are formed at the crack convergence stage, indicating that the concrete structure has to be repaired. Therefore, this study is mainly focused on the statistical evolution of the nucleation and growth of microcracks.

Bai et al. [35] found that the nucleation rate of microcracks, ${\dot{n}}_{N}$, is related to the local stress $\sigma_{t}$ and the crack length *a*, which can be well described as:

(10) $${\dot{n}}_{N}=k_{th}\left(\frac{\sigma_{t}}{\sigma_{th}}-1\right)\left(\frac{a}{a_{th}}\right)\exp\left[-{\left(\frac{a}{a_{th}}\right)}^{m}\right]\;,\;\left(\sigma_{t}>\sigma_{th}\right)$$

where $k_{th}$ and *m* are material constants, $a_{th}$ is the reference length,$\sigma_{th}$ is the threshold stress of microcrack nucleation, and $\sigma_{t}$ is a function of the external load and corrosion factor.

Corrosion can be converted into an internal expansion force *p* through chemical mechanics. Therefore, $\sigma_{t}$ can be expressed as the superposition of an external load and an internal expansion force, that is:

(11) $$\sigma_{t}=\sigma_{0}+\beta_{p}p$$

where $\beta_{p}$ is a proportional coefficient describing the effect of corrosion factor, and $\sigma_{0}$ is stress caused by the external load. The latter can be determined by the second invariant of far-field stress deflection $J{’}_{2(\mathrm{Rem})}$, and we have:

(12) $$\sigma_{0}=\beta_{\sigma }\sqrt{3J{’}_{2(Rem)}}$$

where $\beta_{\sigma }$ is a proportional coefficient.

The internal expansion force, the second term on the right-hand side of Equation (10), is derived by using the chemical reaction rate of delayed ettringite formation. Here, it is assumed that the increase in expansion stress d*p* is proportional to the increase in delayed ettringite concentration,$dC_{\mathrm{Ettringite}}$, namely:

(13) $$dp\propto dC_{\mathrm{Ettringite}}$$

Then, according to Equations (7) and (13), we can obtain:

(14) $$C_{CA}=-\lambda \left(p-p*\right)$$

where λ and p* are the parameters to be determined.

Substituting Equation (14) into Equation (6), we have:

(15) $$\frac{d\left(p-p*\right)}{dt}=-k\beta_{p}\left(p-p*\right)$$

Solving Equation (15) for p, we obtain:

(16) $$p=p^{*}[1-\exp(-t/t_{p})]$$

where *t_p_* is the characteristic time.

Based on the results obtained by Seaman et al. [38] and Stenvens et al. [39], the microcrack growth equation is derived as:

(17) $$\begin{array}{l} \dot{a}=g(t)a\\ g(t)=\left(\sigma_{t}-\sigma_{th}\right)/4\eta \end{array}$$

where η is the viscosity coefficient.

Based on the number balance principle of microcracks between [*a*, *a*+d*a*], a partial differential equation is obtained [35], that is:

(18) $$\frac{\partial n}{\partial t}+\frac{\partial (\dot{a}n)}{\partial a}={\dot{n}}_{N}$$

Substituting Equation (17) into Equation (18) yields:

(19) $$\frac{\partial n}{\partial t}+\frac{\partial n}{\partial a}\dot{a}+g(t)={\dot{n}}_{N}$$

Such a first-order partial differential equation can be transformed into a first-order ordinary differential equation on the characteristic line. Then, the evolution equation of the number density of microcracks can be written as:

(20) $$\frac{dn}{dt}+g(t)n={\dot{n}}_{N}$$

Multiplying both sides of Equation (20) by $\beta_{a}a^{3}$ and integrating it gives:

(21) $$\int_{0}^{\infty }\beta_{a}a^{3}\frac{dn}{dt}da+\int_{0}^{\infty }g\left(t\right)n\beta_{a}a^{3}da=\int_{0}^{\infty }\dot{n_{N}}\beta_{a}a^{3}da$$

From Equation (9), it is seen that Equation (21) can be rewritten as:

(22) $$\frac{dD}{dt}+g\left(t\right)D=Q\left(t,a\right)$$

(23) $$Q\left(t,a\right)=\int_{0}^{\infty }{\dot{n}}_{N}\beta_{a}a^{3}da$$

Solving Equation (22) and noting $D{|}_{t=0}=0$, the damage evolution under sulfate attack and external loading can be obtained as:

(24) $$D=e^{-\int g\left(t\right)dt}\int_{0}^{t}Q\left(\tau ,a\right)\;e^{\int g\left(t\right)dt}d\tau$$

If the effect of remote loads is not taken into account, the parameter $\beta_{\sigma }$ in Equation (12) should be zero.

## 3. Experimental

**Figure 3:**
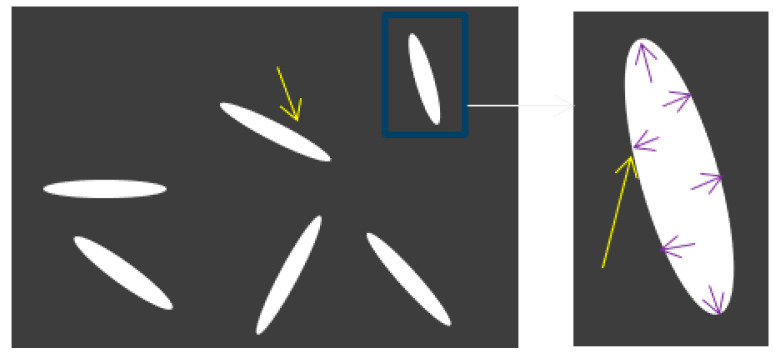
Schematic representation of concrete after corrosion, where the loading condition is shown in a partially enlarged crack (right).

To detect the evolution of corrosion damage expressed by Equation (24), concrete can be approximately regarded as a composite composed of a concrete matrix and corrosion cracks. As illustrated in Figure 3, there is an expansive force in corrosion cracks.

The concrete modulus after corrosion, *E*, is determined by means of the volume average, that is:

(25) $$E=\frac{1}{V}\int_{V}E_{0}dV=\frac{V-V_{C}}{V}E_{0}=\left(1-\frac{V_{C}}{V}\right)E_{0}$$

where *V* and *V_C_* are the volume of concrete and the total volume of corrosion cracks, and *E*_0_ is the modulus of concrete matrix (or concrete before corrosion).

Note that *V_C_*/*V* is the volume occupied by cracks per unit volume, which is equal to the corrosion damage, *D*. Hence, Equation (25) can be written as:

(26) $$D=1-\frac{E}{E_{0}}$$

### 3.1. Experimental Materials and Sample Preparation

Po42.5 cement, produced by Yangzhou Lvyang Cement Co. Ltd., Yangzhou, China, was applied for the preparation of test specimens. The sodium sulfate solution for corrosion was prepared manually. Analytical grade pure sodium sulfate (from Shanghai Chemical Reagent Company of China Pharmaceutical (Group)) was used with a molecular weight of 142.04.

The water-to-cement ratio has a significant influence on the mechanical properties of cement mortar. Therefore, mortar specimens with different water-to-cement ratios were prepared to determine their relationships with the mechanical properties of mortar. As listed in Table 1, standard specimens with dimensions of 150 × 150 × 150 mm^3^ were used to determine the dynamic elastic modulus, and specimens with dimensions 10 × 10 × 70 mm^3^ were used for SEM tests to observe microstructures of concrete specimens.

### 3.2. Experimental Scheme

Cement mortar specimens were kept under the environmental conditions of 20 °C and 90% humidity for 24 h and, then, were transferred to a standard curing room for 28 d after removing formworks. These specimens were evenly divided into three groups and soaked sodium sulfate [$SO_{4}^{2-}$] solutions with three different concentrations of 0%, 3%, and 8%, to detect the dynamic elastic moduli of specimens on 14 d, 28 d, 60 d, 90 d, …, and 450 d, respectively.

### 3.3. Testing Method of Dynamic Elastic Modulus

**Figure 4:**
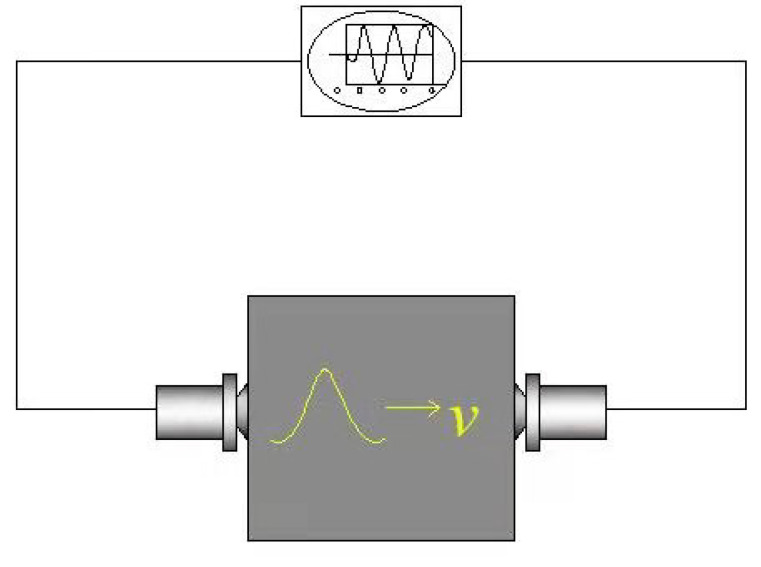
Illustration of an ultrasonic testing method of dynamic elastic modulus.

The test method of the dynamic elastic modulus consists of several steps. First, a relatively complete surface of a sample was taken as a benchmark. Then, ultrasonic wave velocities at five points were measured at four points near the vertex and one point in the middle, as illustrated in Figure 4, in which, the symbol “*v*” denotes the ultrasonic wave speed. Finally, the average value of five points was reported.

## 4. Results and Discussion

### 4.1. Experimental Results

**Figure 5:**
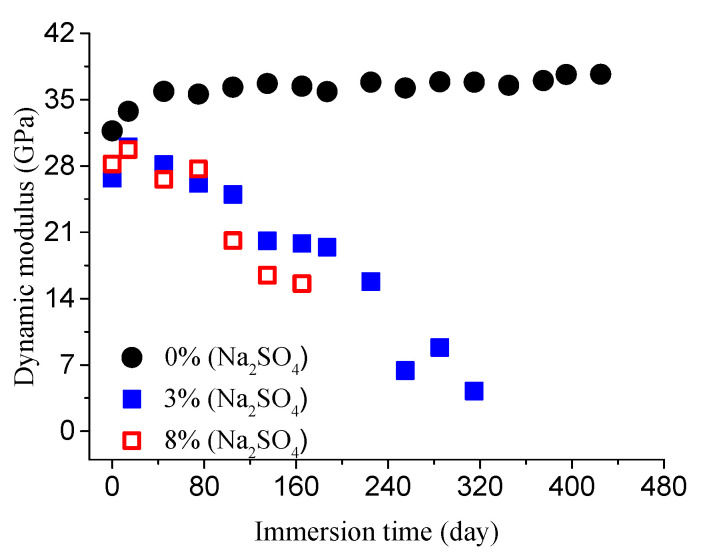
Dynamic modulus evolution of concrete specimens with the water-to-cement ratio of 0.4 in different concentrations of sodium sulfate solution.

**Figure 6:**
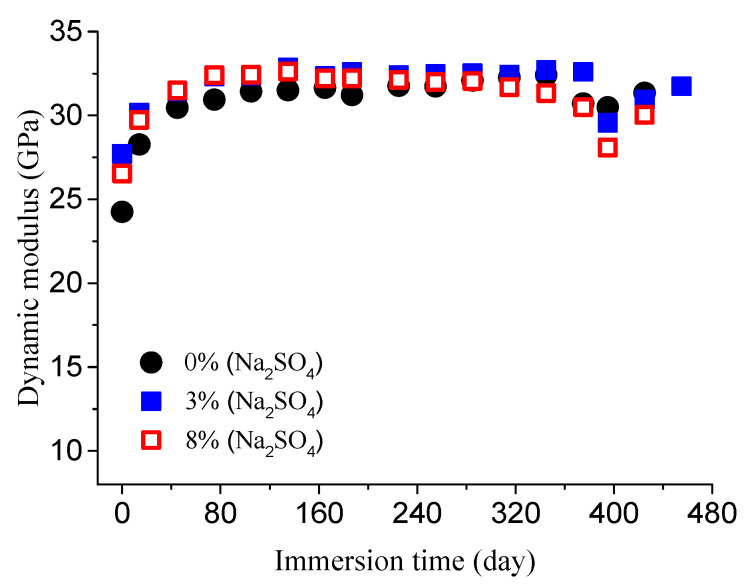
Dynamic modulus evolution of concrete specimens with the water-to-cement ratio of 0.7 in different concentrations of sodium sulfate.

The dynamic elastic modulus was measured at several intervals. The obtained results are shown in Figure 5 and Figure 6.

As you can see from Figure 5 and Figure 6, the increase in the modulus is due to continuous hydration of concrete and the filling effect of delayed ettringite, which results in negligible concrete damage. With the increase in corrosion time, the modulus begins to decrease. This is due to the corrosion damage evolution.

### 4.2. Experimental Determination of Corrosion Damage Model Parameters

**Figure 7:**
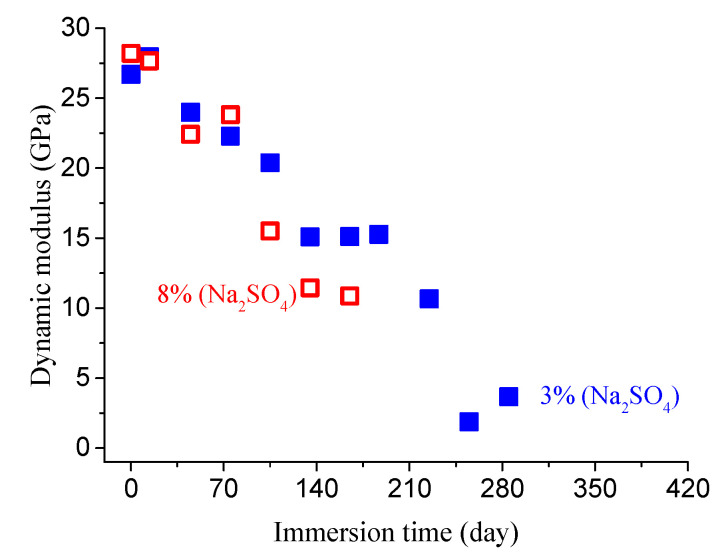
Dynamic modulus evolution of concrete specimens (*w*/*c* = 0.4) after eliminating hydration in different sulfate solutions.

**Figure 8:**
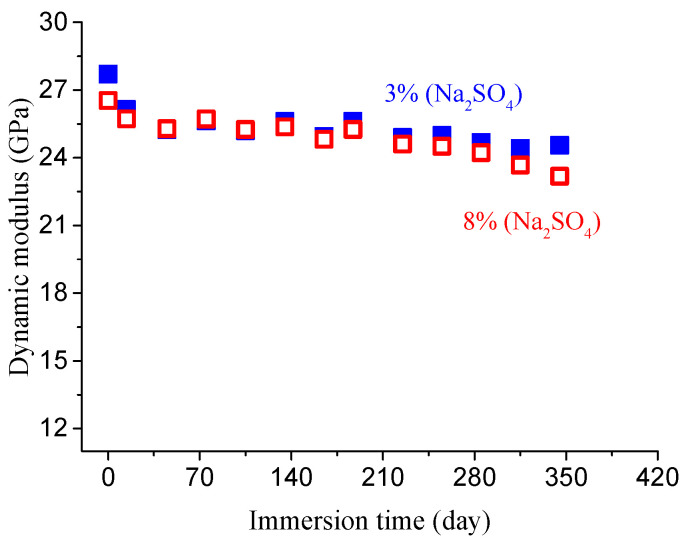
Dynamic modulus evolution of concrete specimens (*w*/*c* = 0.7) after eliminating hydration in different sulfate solution.

By neglecting the effect of continuous hydration, the evolution of the concrete dynamic modulus can be determined, as shown in Figure 7 and Figure 8.

**Figure 9:**
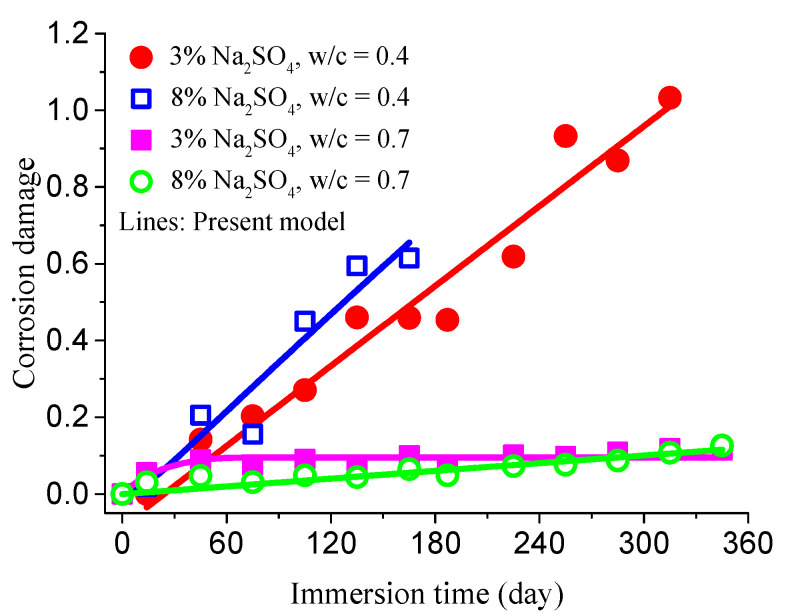
Damage evolution results of concrete specimens.

Based on these experimental results, the concrete damage evolution under sulfate attack can be obtained by Equation (26), as plotted in Figure 9.

From Equation (10) and Equation (23), we have:

(27) $$\begin{array}{l} Q(t,a)=\int_{0}^{\infty }{\dot{n}}_{N}\beta_{a}a^{3}da=\int_{0}^{\infty }k_{th}\beta_{a}\left(\frac{\sigma_{t}}{\sigma_{th}}-1\right)\left(\frac{a}{a_{th}}\right)\exp\left[-\left(\frac{a}{a_{th}}\right)\right]a^{3}\;da\\ =24k_{th}\beta_{a}\left(\frac{\sigma_{0}+P_{0}^{*}\left[1-\exp(-t/t_{p})\right]}{\sigma_{th}}-1\right) \end{array}$$

Substituting Equation (11) into Equation (17) gives:

(28) $$g\left(t\right)=\frac{\sigma_{0}+P_{0}^{*}\left[1-\exp\left(-t/t_{p}\right)\right]-\sigma_{th}}{4\eta }$$

Therefore, we can obtain:

(29) $$\int g\left(t\right)\;dt=\frac{\sigma_{0}-\sigma_{th}}{4\eta }t+\frac{P_{0}^{*}}{4\eta }t+\frac{\exp(-t/t_{p})P_{0}^{*}t_{p}}{4\eta }$$

By substitution of Equations (29) and (27) into Equation (24), the expression of damage evolution can be derived under the simultaneous action of external load and corrosion factors. Considering the damage evolution of concrete only under sulfate, i.e., $\sigma_{0}=0$, we have:

(30) $$\begin{array}{l} D=\frac{96\eta k}{\sigma_{th}}\left[\varsigma +\exp\left(\psi \left(t\right)\right)\right]\cdot \exp\left(-\psi \left(t\right)\right)\\ k=k_{th}\beta_{a},\;\varsigma =-\exp\left(P_{0}^{*}t_{p}/4\eta \right)\;\\ \psi \left(t\right)=\left(P_{0}^{*}t_{p}\cdot \exp\left(-t/t_{p}\right)+\left(P_{0}^{*}-\sigma_{th}\right)\;t\right)/4\eta \end{array}$$

Here, the threshold stress $\sigma_{th}$= 4 MPa of microcrack nucleation was determined with the design value of axial tensile strength of concrete. Using Equation (30), the specific fitting results are shown in Figure 9.

It is seen that experimental results are consistent with those obtained from the damage model. When concrete specimens are exposed to sulfate attack, the internal damage evolution law of concrete is also in agreement with the model given by Equation (30). Therefore, such a modified model can be applied to characterize the internal damage evolution law of concrete.

## 5. Conclusions

It was theoretically proven that the increase in the space occupied by corrosion cracks per unit volume is equivalent to the reduction of the concrete modulus due to corrosion. Therefore, it is reasonable to use ultrasonic technology to detect changes in concrete modulus to analyze damage evolution.

(1) By combining the equilibrium equation of microcrack number density evolution and the chemical reaction rate equation, the first-order partial differential equations were derived. The corrosion damage evolution model of concrete under sulfate attack was developed by integrating the volume of microcrack number density.
(2) The new corrosion damage model established in this paper is able to accurately describe the internal damage evolution process of concrete under sulfate corrosion. The analysis method proposed in this paper provides a reference for the corrosion damage analysis of other materials.

## Figures and Tables

**Table 1:** Mix proportions of mortar, where H and Z indicate specimens for dynamic tests and microstructural observation, respectively.

Sample	Cement	Sand	*w*/*c*
H1	1	3	0.4
H3	1	3	0.7
Z	1	2	0.45
